# Morphology-based ECG criteria for identifying left-atrial low-voltage areas on voltage mapping: a diagnostic accuracy study

**DOI:** 10.3389/fcvm.2026.1891500

**Published:** 2026-08-13

**Authors:** Ruben Hoffmann, Pieter Koopman, Laurens Verhaeghe, Jochen Van Wabeke, Johan Vijgen, Nathalie Antole, Joris Schurmans, Dagmara Dilling-Boer, Thomas Phlips

**Affiliations:** 1 LCRC(-MHU), Faculty of Medicine and Life Sciences, UHasselt, Diepenbeek, Belgium; 2 Department of Cardiology, Jessa Hospital, Hasselt, Belgium; 3 Department of Jessa & Science, Jessa Hospital, Hasselt, Belgium; 4 Department of Cardiology, Centre Hospitalier Chrétien MontLégia, Liège, Belgium

**Keywords:** atrial fibrillation, ECG criteria, left atrial fibrosis, low-voltage areas, pulmonary vein isolation (PVI), P-wave morphology, voltage mapping

## Abstract

**Background:**

Left atrial (LA) fibrosis contributes to atrial fibrillation (AF) recurrence beyond pulmonary vein triggers, yet its non-invasive identification pre-ablation remains challenging. While invasive voltage mapping may identify low-voltage areas (LVAs) as a surrogate of atrial substrate abnormality, it is impractical for systematic screening.

**Objective:**

This single-center retrospective pilot study evaluated the diagnostic performance of predefined ECG morphological criteria for detecting LVAs.

**Methods:**

LVAs were used as a surrogate marker for atrial fibrosis and defined as bipolar voltage of <0.50 mV on invasive mapping in sinus rhythm or CS pacing. Four ECG criteria were evaluated: (1) history of atypical atrial flutter, (2) negative atrial vector attenuation in V1, (3) presence of typical or atypical advanced interatrial block (types I–IV), and (4) poor terminal P-wave progression in V4–V6. The presence of at least one of these criteria was considered indicative of LVAs. Diagnostic performance of the ECG criteria was assessed against invasive voltage mapping as the reference standard. Five electrophysiologists independently analyzed 12-lead ECGs (100 mm/s, 40 mm/mV) from 78 patients undergoing first-time AF ablation.

**Results:**

LVAs were present in 35 patients. The predefined ECG-based criteria demonstrated sensitivity 85.7% (95% CI: 70.6–93.7), specificity 95.4% (95% CI: 84.5–98.7), positive predictive value 93.8% (95% CI: 79.9–98.3), and negative predictive value 89.1% (95% CI: 77.0–95.3), with an overall diagnostic accuracy of 91.0% (95% CI: 82.6–95.6) and an AUC of 0.905 (95% CI: 0.838–0.972). Interatrial block and poor terminal P-wave progression were the most frequent predictors.

**Conclusions:**

This proof-of-concept study demonstrated promising diagnostic performance for morphological ECG-criteria in detecting left-atrial LVAs. The proposed four-criteria model correlated strongly with invasive voltage mapping and may represent a promising candidate for further validation as a non-invasive tool for pre-procedural identification of atrial substrate.

## Introduction

Atrial fibrillation (AF) is the most prevalent sustained cardiac arrhythmia and represents a major cause of morbidity and mortality worldwide (1). Pulmonary vein isolation (PVI) is the standard invasive treatment, stemming from the recognition of the pulmonary veins as a primary source of AF (2, 3). Although success rates have slowly risen in the last decades, recurrence after ablation is still prevalent. This can partly be attributed to PVI reconnections due to incomplete PVI, although this number is declining (4, 5).

An increasing reason for AF recurrence is located outside of the pulmonary veins (3). Known substrates or triggers include the cavotricuspid isthmus-dependent flutter, the vein of Marshall and the superior vena cava (3). Finally, fibrotic tissue may lead to conduction heterogeneity and delayed activation (6–8), creating a substrate for re-entry and arrhythmia persistence. Invasive electroanatomic voltage mapping remains the current clinical reference standard for identifying areas of low-voltage areas (LVAs). However, this technique is technically demanding, time-consuming and associated with non-negligible economic costs, including extra high-density mapping catheters and catheterization laboratory time. This makes it impractical for universal screening. Therefore, the development of a simple, non-invasive method for detecting LVAs could be of significant clinical value. Pre-procedural identification of LVAs may help guide procedural planning, including phenotyping of patients that may benefit from adjunctive substrate modification which in turn may support more individualized patient selection prior to ablation.

Since the surface electrocardiogram (ECG) reflects global atrial depolarization, subtle alterations in P-wave morphology can directly mirror the presence of conduction delay caused by fibrotic remodeling. Previous studies have associated P-wave features such as advanced interatrial block (A-IAB), P-wave duration and P-wave dispersion with AF recurrence after ablation (9–12). Building upon these concepts, we hypothesized that specific ECG morphological characteristics could predict the presence of LVAs in patients with AF.

The present pilot study aims to test predefined morphological ECG criteria for their ability to identify LVAs, using invasive LA voltage mapping as the reference standard.

## Methods

### Study population

**Figure 1:**
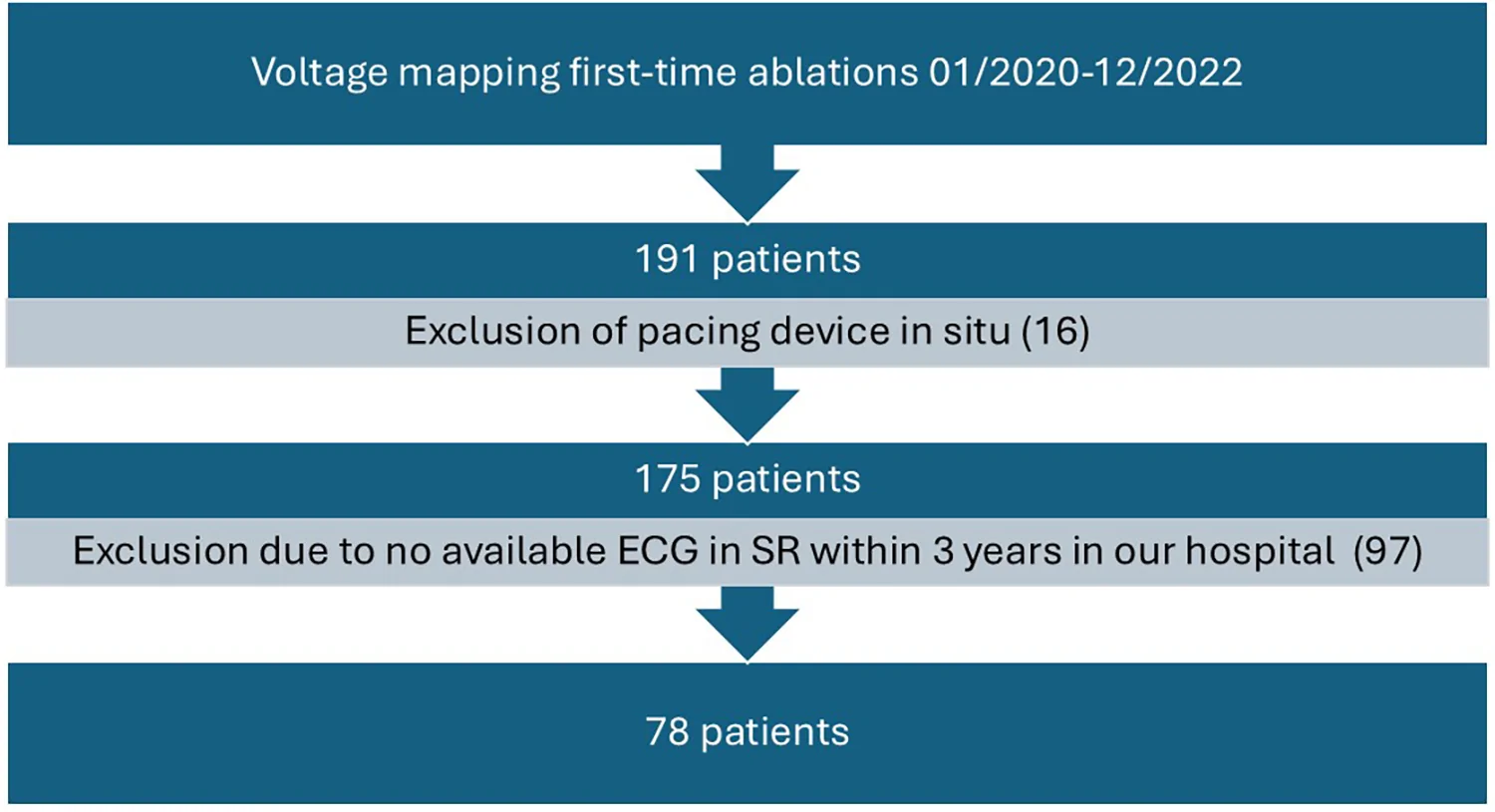
Exclusion of patients.

This retrospective observational study analyzed data from patients undergoing first-time PVI between January 2020 and December 2022. Patients were eligible if a LA voltage map was available and if a 12-lead sinus rhythm (SR) ECG, performed at our institution within three years before ablation, was available. The closest ECG in SR to the ablation date was used. All patients received voltage mapping using the CARTO 3 mapping system and were retrospectively evaluated using both CARTO and CARTOnet (13). Patients were excluded if the patient had an implantable device with any pacing capabilities (both atrial or ventricular). An initial 191 patients undergoing first-time AF ablation using CARTO voltage mapping were screened. After exclusion, 78 patients remained eligible for analysis (Figure 1). As this was a hypothesis-generating pilot study, no formal *a priori* sample size calculation was performed; the final sample size was determined pragmatically by the number of patients meeting eligibility criteria within the study period.

The study was performed in compliance with the principles outlined in the declaration of Helsinki and approved by the Institutional Ethics committee.

### Mapping procedure

All procedures were performed under general anesthesia. Catheter choice and ablation strategy depended on operator preference and patient-specific factors. Patients were ablated using the SmartTouch (Biosense Webster Inc.) (*N* = 40), QDOT (Biosense Webster, Inc.) (*N* = 28) or Heliostar (Biosense Webster, Inc.) (*N* = 10) catheter. Five different mapping catheters were used: the SmartTouch (*N* = 31), the QDOT (*N* = 21) the LASSOSTAR (Biosense Webster, Inc.) (*N* = 10), the PENTARAY (Biosense Webster, Inc.) (*N* = 15) and the OCTARAY (Biosense Webster, Inc.) catheter (*N* = 1). Voltage maps were made with the wavefront algorithm in sinus rhythm or during proximal CS pacing with a threshold for contact (more or equal to 4 g) or using the tissue proximity indication (TPI) feature when available.

### Definition of LVAs

LVAs on invasive bipolar voltage mapping, with amplitudes <0.50 mV, were used as a surrogate marker of atrial fibrosis (14). To quantify substrate burden, the proportion of the left atrial surface area demonstrating low voltage was calculated. For analytical purposes, patients were dichotomized using a 5% threshold of low-voltage area relative to the total mapped atrial surface area, classifying patients as either LVA-negative (<5%) or LVA-positive (≥5%). No universally accepted threshold exists for dichotomizing low-voltage burden. The 5% cutoff was therefore chosen *a priori* as a pragmatic threshold approximating previously described staging systems derived from MRI-based atrial fibrosis classifications, and based on previous studies allowing for a conservative dichotomization of minimal vs. more extensive substrate (15, 16).

LVA burden was quantified using the CARTOnet platform, which incorporates an automated tool for calculating the proportion of low-voltage surface area relative to the total mapped left atrial surface. The pulmonary veins and mitral valve annulus were excluded from all analyses. In patients undergoing radiofrequency ablation, voltage mapping was performed after PVI, with the radiofrequency ablation points defining the pulmonary vein exclusion boundary. In patients treated with an alternative ablation modality, voltage mapping was performed prior to the ablation procedure. In addition to this automated tool, all voltage maps were assessed by consensus between three experienced electrophysiologists as being LVA positive or not, blinded to patient identification data, ECG findings, and clinical characteristics.

It should be noted that bipolar LVAs are an electrophysiological surrogate of atrial fibrosis rather than a direct histological measure. Voltage amplitudes are influenced by several factors including rhythm, wavefront direction, catheter type, contact force, and mapping density. Accordingly, throughout this manuscript, LVAs should be interpreted as a marker of abnormal electro-anatomical substrate rather than confirmed fibrosis.

### Electrocardiographic acquisition and analysis

Standard 12-lead ECGs were obtained prior to the ablation procedure. All ECGs were obtained using the SEMA data framework (Schiller) for ECGs. For detailed P-wave morphology analysis, ECGs were amplified (100 mm/s paper speed and 40 mm/mV amplitude).

**Figure 2:**
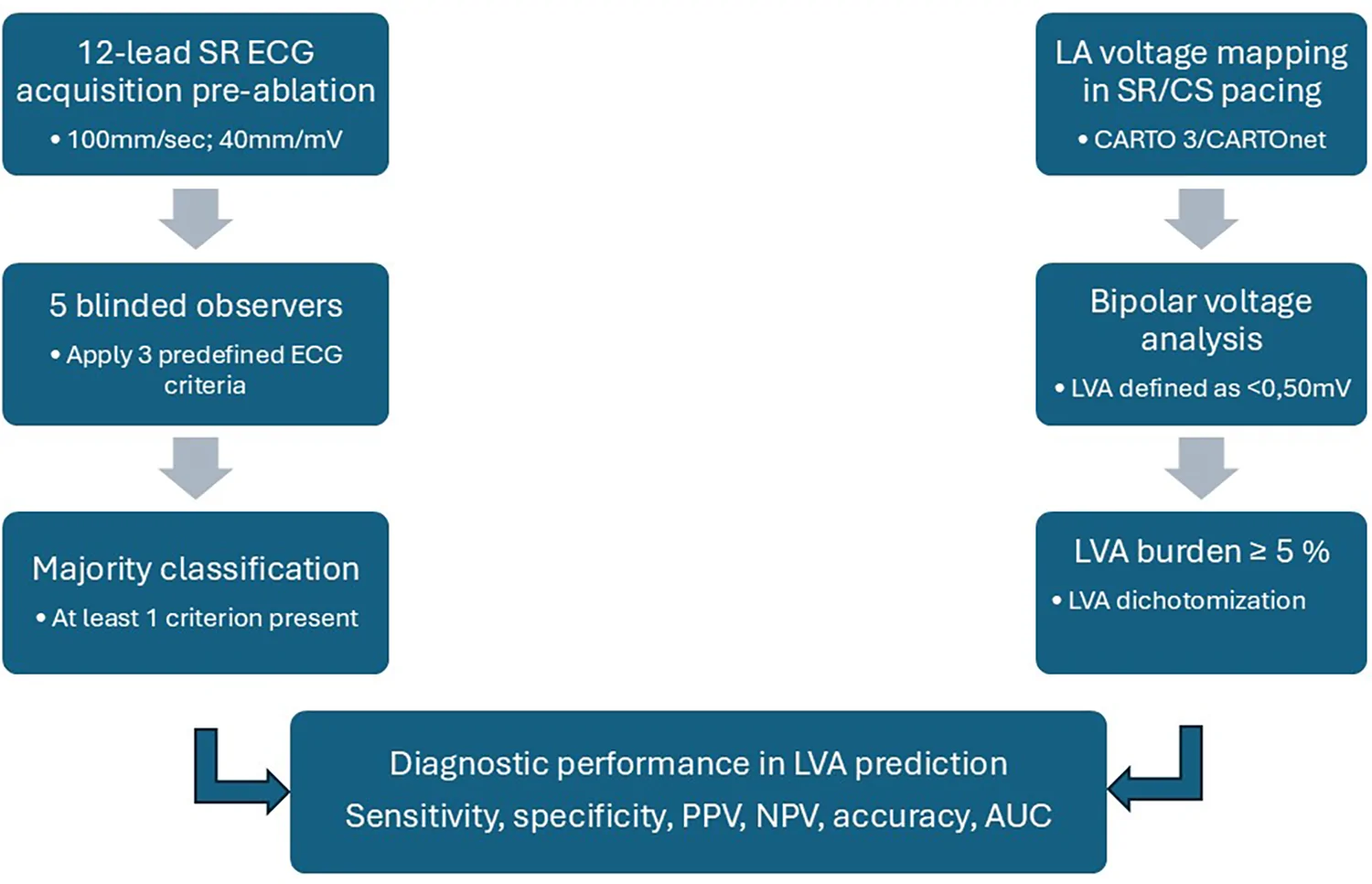
Study workflow.

**Figure 3:**
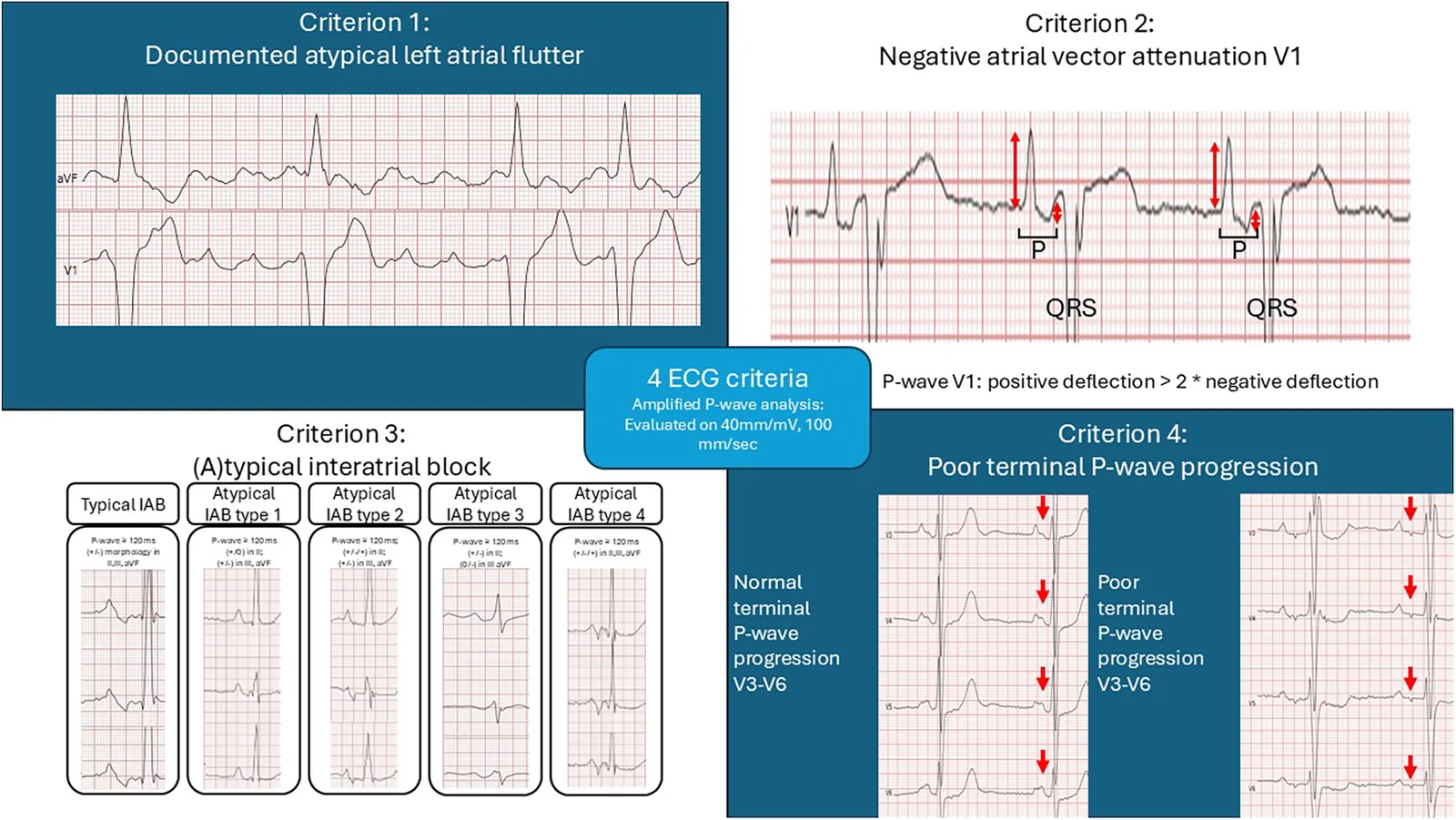
Description of the 4 ECG criteria.

The presence of at least one criterion was considered predictive of LVAs. To evaluate interobserver variability, three electrophysiologists and two electrophysiologists in training, blinded to mapping results and each other's conclusions, analyzed all ECGs. Observers received no formal preliminary training and only received Figure 3 for reference. Final classifications were determined by majority decision among the five observers. Primary diagnostic performance analysis was based on majority classification, with individual observer performance assessed separately. The diagnostic performance of the ECG-based criteria was evaluated against invasive mapping, regarded as the reference standard. The overall study design is summarized in Figure 2. Sensitivity, specificity, positive predictive value (PPV), negative predictive value (NPV), and overall accuracy were calculated. This performance was also set out against commonly known clinical risk scores including the CHA_2_DS_2_-VA score, the APPLE score and the DR-FLASH score (17–19). Lastly, the performance of P-wave duration was evaluated. Formal statistical comparison of AUC values between the proposed ECG criteria and comparator scores was not performed given the exploratory nature of this pilot study and the limited sample size, which would render such comparison underpowered.

### Statistical analysis

All statistical analyses were performed with the R statistical software (The R Foundation for Statistical Computing, Vienna, Austria; version 4.3.3). Categorical variables are presented as frequencies and percentages, while continuous variables as means ± standard deviation or median with interquartile ranges, as appropriate. There were no missing data for any variable included in the analyses. Chi squared test or the Fisher exact test were used to test for differences in medication use. Normality was assessed using the Shapiro–Wilk test. The reproducibility of the criteria was set as the level of agreement between investigators in determining the presence of LVAs using Conger's Kappa. The optimal cut-off for P-wave duration was determined using Youden's index, with sensitivity analyses at 140 and 150 ms. To assess whether the proposed ECG criteria remained independently associated with LVAs after adjustment for relevant clinical variables, a multivariable analysis was performed using Firth's penalized logistic regression. This method was selected as the recommended approach for small samples prone to complete or near-complete separation. This multivariable analysis was not part of the pre-specified primary analysis plan and was performed as an exploratory, hypothesis-generating analysis to evaluate whether the association between the ECG criteria and LVAs persisted after adjustment for relevant clinical variables. Variables included in the model were: ECG criteria majority classification, obesity, LVEF, heart failure, LA volume, AF type, and antiarrhythmic drug use. OR estimates are reported for completeness but are prone to instability and should not be interpreted as reliable effect size estimates. *P*-values are considered the more meaningful metric in this context. A *p*-value ≤0.05 was considered statistically significant.

### Criteria description

Based on preliminary observation and pathophysiological rationale, four pre-specified criteria were established combining four morphological ECG criteria (Figure 3):

- Documentation of atypical left-sided atrial flutter.

As atypical left-sided atrial flutter often arises from re-entrant circuits that depend on areas of diseased atrial tissue, this criterion was taken as the first indicator of LVAs. Documentation of atypical left-sided atrial flutter was adjudicated based on explicit mention in the medical history. A general diagnosis of atrial flutter was not considered sufficient for this criterion.

- Negative vector attenuation in V1 defined as:
  ○ Positive-to-negative amplitude ratio >2 in the P-wave of lead V1.

**Figure 4:**
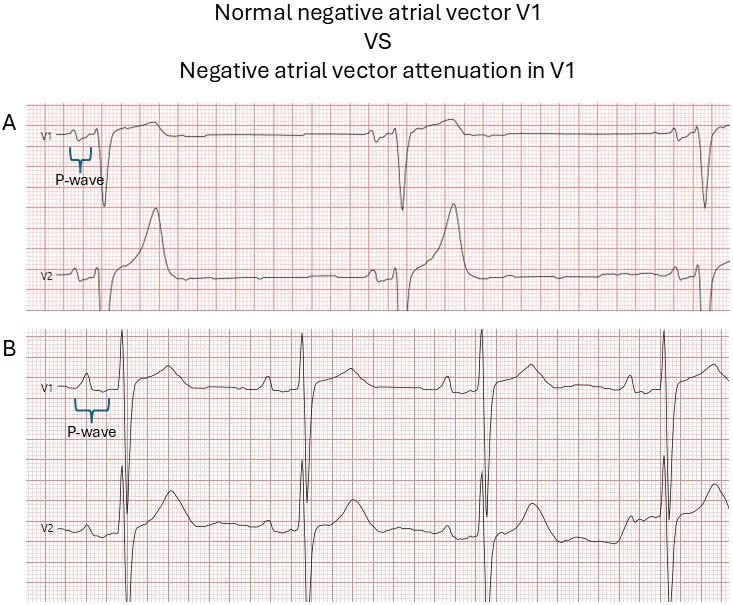
An example of negative atrial vector attenuation. leads V1–V2 of a 12-lead ECG (40 mm/mV, 50 mm/s) in Sinus rhythm before first-time ablation. The baseline is defined as the isoelectric point immediately preceding the P-wave onset. The positive amplitude is measured as the maximum peak deflection above baseline, and the negative amplitude as the maximum peak deflection below baseline, both measured from this same pre-P-wave baseline. In cases where multiple deflections are present, the largest amplitude in each direction is used for the ratio calculation. **(A)** A normal negative atrial vector in a 70-year old patient with persistent AF, moderate dilatation, no significant low-voltage areas on invasive voltage mapping. **(B)** Negative atrial vector attenuation in a 76-year old patient with persistent AF, mild dilatation, diffuse low-voltage areas.

Under normal conditions, atrial depolarization produces a balanced biphasic P wave in lead V1 with an initial positive component reflecting right atrial depolarization, followed by a negative component corresponding to left atrial activation (Figure 4A). LA dilatation is typically associated with a deep terminal negative component in V1 and slight prolongation of the P wave. In patients with LVAs, the contribution of this vector may be attenuated due to conduction slowing and reduced electrical voltage within fibrotic regions. This may result in relative dominance of the initial right atrial component. To capture this imbalance between right and left atrial activation vectors, the ratio of positive and negative amplitudes was evaluated. A ratio greater than 2 was selected before data collection as a pragmatic threshold indicating attenuation of the LA component. Typically, this results in a P-wave in V1 with an initial peaked short positive deflection followed by a shallow lengthy P-wave with an additional small late negative deflection (Figure 4B).

The threshold of >2 for the positive-to-negative amplitude ratio in V1 was selected empirically based on prior observation of P-wave morphology and was pre-specified before data collection. Nearby cutoffs were not formally examined in this pilot study.

- Presence of advanced typical or atypical interatrial block (types I–IV).

Advanced interatrial block has been linked to atrial fibrillation and to LVAs which presents mostly antero-superior (region 4b) (20) at the insertion of the Bachmann bundle (9, 21).

- Poor terminal P-wave progression in leads V4–V6, defined as either:
  ○ a negative segment in the second half of the P-wave in at least two leads in V4–V6

OR

○ an iso-electric segment in the second half of the P-wave in at least two leads in V4–V6.

**Figure 5:**
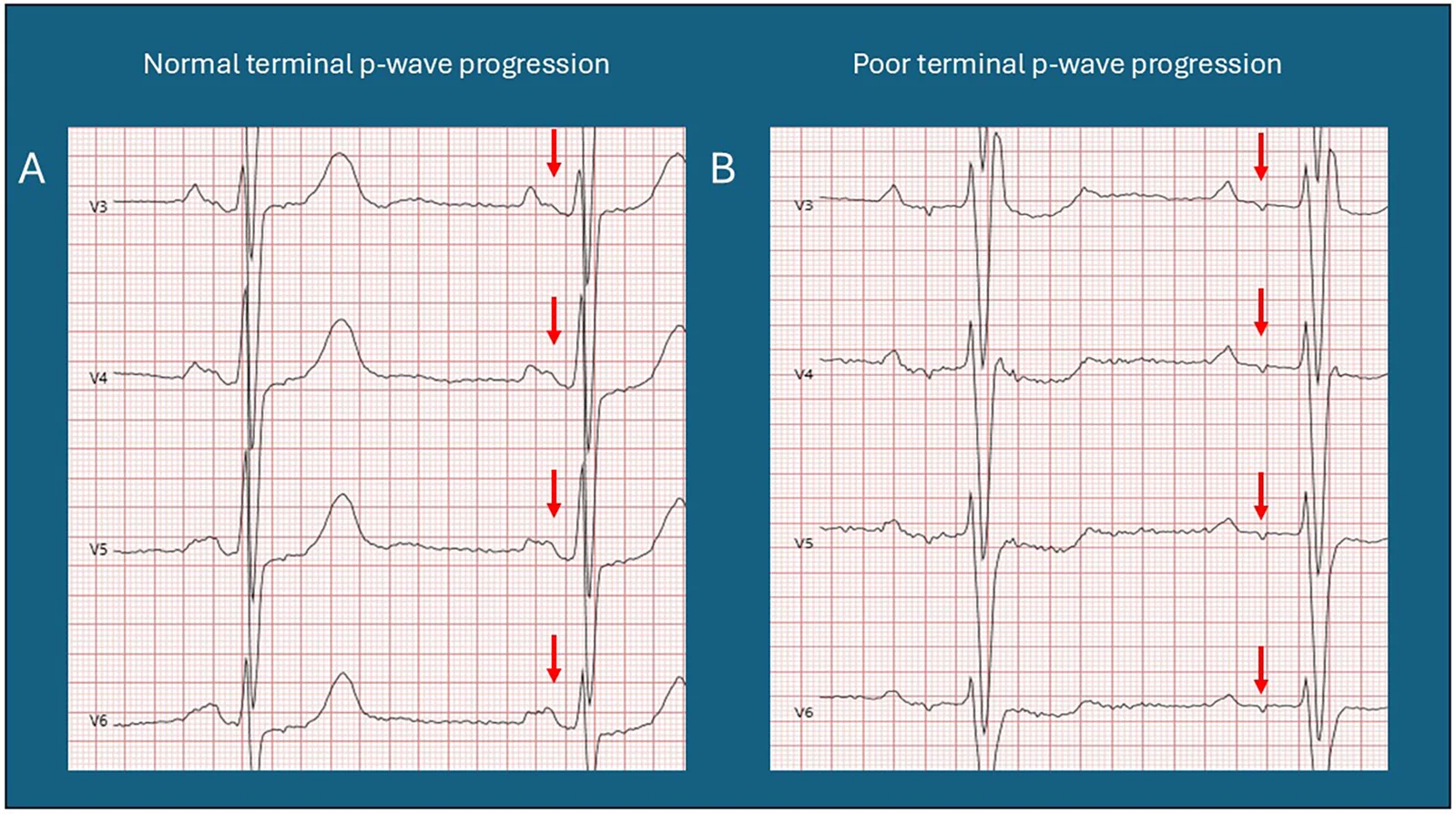
An example of poor terminal P-wave progression in leads V4–V6, defined as either a negative or iso-electric segment in the second half of the P-wave duration in at least two of leads V4–V6. The second half is defined temporally as the latter 50% of total P-wave duration, measured from P-wave onset to offset. leads V3–V6 of a 12-lead ECG (40 mm/mV, 50 mm/s) in Sinus rhythm before first-time ablation. **(A)** Normal terminal P-wave progression V4–V6 in a 69 year-old patient, persistent AF, mild left atrial dilatation, low-voltage areas <5%. **(B)** Poor terminal P-wave progression V4–V6 in a 83 year-old patient, persistent AF, mild left atrial dilatation, diffuse low-voltage areas.

Normal atrial depolarization results in a terminal P-wave which becomes more positive as one goes through the precordial leads (Figure 5A). We hypothesize that LVAs may result in poor evolution in the terminal P-wave over the lateral leads (Figure 5B).

## Results

### Patient characteristics

Among the 78 patients included, 35 (13 with paroxysmal AF and 22 with persistent AF) demonstrated LVAs on voltage mapping, while 43 (29 paroxysmal, 14 persistent) did not. There was no difference between the AI tool and the consensus of observers.

A numerically higher proportion of patients with LVAs had diabetes mellitus type 2, heart failure (both HFpEF and HFrEF), obesity, a higher frequency of persistent AF and more dilated LA. Numerical differences in antiarrhythmic drug use were observed, but these did not reach statistical significance (Table 1).

**Table 1:** Baseline characteristics.

Categorical	All	%	no LVAs	%	LVAs	%	
	78		43		35		
AHT	48	61.5%	28	65.1%	20	57.1%	
DM	10	12.8%	4	9.3%	6	17.1%	
HF	30	38.5%	14	32.6%	16	45.7%	
LAV, normal	19	24.4%	15	34.9%	4	11.4%	
LAV, mild	17	21.8%	13	30.2%	4	11.4%	
LAV, moderate	11	14.1%	3	7.0%	8	22.9%	
LAV, severe	31	39.7%	12	27.9%	19	54.3%	
OSAS	8	10.3%	2	4.7%	6	17.1%	
TIA.CVA	6	7.7%	5	11.6%	1	2.9%	
Familial history	31	39.7%	19	44.2%	12	34.3%	
Sex, M	52	66.7%	31	72.1%	21	60.0%	
Lung disease	9	11.5%	5	11.6%	4	11.4%	
Obesity	33	42.3%	16	37.2%	17	48.6%	
Smoking, stopped	19	24.4%	10	23.3%	9	25.7%	
Smoking, active	7	9.0%	3	7.0%	4	11.4%	
Type AF, paroxysmal	42	53.8%	29	67.4%	13	37.1%	
Vascular disease	19	24.4%	8	18.6%	11	31.4%	
Mapping rhythm SR	49	62.8%	31	72.1%	18	51.4%	

Medication	All	%	no LVAs	%	LVAs	%	*p*-value
Beta blocker	48	61.5%	29	67.4%	19	54.3%	0.627
Class 1c	19	24.4%	13	30.2%	6	17.1%	0.587
Amiodarone	26	33.3%	12	27.9%	14	40.0%	0.376
Diltiazem	2	2.6%	2	4.7%	0	0.0%	0.499
Sotalol	9	11.5%	3	7.0%	6	17.1%	0.298
Verapamil	2	2.6%	1	2.3%	1	2.9%	1.000

Continuous	All (Median/mean)	SD/IQR	no LVAs (Median/mean)	SD/IQR	LVAs (Median/mean)	SD/IQR	
GFR (normal)	69	21.8	75.4	20.7	61.1	20.7	
LVEF (non-normal)	60	55–60	60	59–60	60	52–62.5	
Age (normal)	68.1	9.71	65.5	10	71.2	8.45	
ECG-to-ablation interval	113	70–236	116	58–192	110	78–252	
P-wave duration (normal)	146	23.7	136	18.7	158	23.3	
Voltage points	384	210–1,105	347	192–598	431	246–1,636	
Percentage LVAs	34	26.50–62.5	NA	NA	34	26.50–62.5	

**Figure 6:**
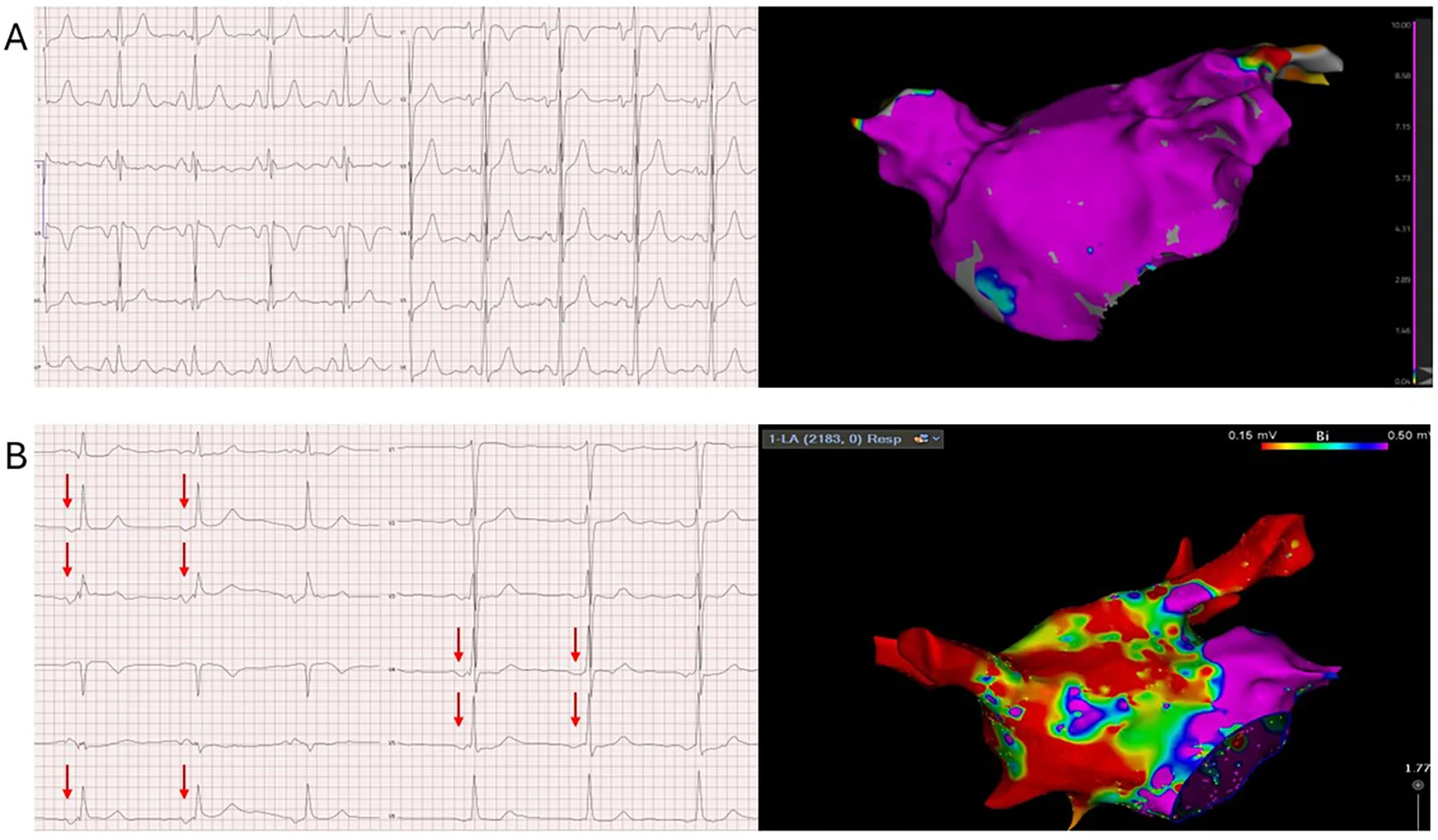
Two representative cases illustrating the relationship between 12-lead ECG morphology and invasive left atrial voltage mapping. For each case, the 12-lead ECG (40 mm/mV, 50 mm/s, recorded in sinus rhythm before first-time ablation) is shown on the left, with the corresponding invasive bipolar voltage map (CARTO 3) on the right. Purple indicates normal bipolar voltage (>0.50 mV); colors approaching red and yellow indicate progressively lower voltages. **(A)** All criteria negative in a 57-year old patient with paroxysmal AF, mild left atrial dilatation. On invasive voltage mapping no significant low-voltage areas. **(B)** presence of advanced interatrial block and poor terminal p-wave progression (red arrows) in a 62-year old patient, persistent AF, severe left atrial dilatation, low voltage areas mostly around the anterior wall.

ECGs were obtained at a median of 113 days (range: 14–1,076) with twelve patients (15%) having ECGs obtained more than 12 months before ablation, of which 10 were taken within 2 years and 2 within 3 years. Among LVA-positive patients, the median LVA was 34.0% of the total mapped left atrial surface area with 17 out of 35 patients exceeding 35%. Representative cases for ECG negative and ECG positive criteria and their corresponding voltage map can be found in Figure 6.

### Criteria performance

The proposed ECG criteria demonstrated strong diagnostic accuracy (AUC: 0.905 (95% CI 0.838–0.972) for identifying LVAs in this pilot cohort (Table 2). All individual criteria showed high specificity and PPV >90%. Atypical atrial tachycardia performed worst [AUC: 0.617 (95% CI 0.540–0.694)] while the remaining three criteria performed relatively similar with AUCs ranging from 0.717 to 0.788. When criterion 1 (history of atypical atrial flutter) was excluded, the three morphological criteria alone demonstrated an AUC of 0.831 (95% CI 0.750–0.913), with sensitivity 68.6% (95% CI 52.0–81.4) and specificity 97.7% (95% CI 87.9–99.6) (Table 3).

**Table 2:** Prediction metrics.

Method		LVA			Value (%)	95% CI
	Test result	Positive	Negative	Metric		
ECG-criteria by majority	Positive	30	2	Sensitivity	85.7	70.6–93.7
	Negative	5	41	Specificity	95.3	84.5–98.7
				PPV	93.8	79.9–98.3
				NPV	89.1	77.0–95.3
				Accuracy	91.0	82.6–95.6
				AUC	0.905	0.838–0.972
APPLE	Positive	25	11	Sensitivity	71.4	54.9–83.7
	Negative	10	32	Specificity	74.4	59.8–85.1
				PPV	69.4	53.1–82.0
				NPV	76.2	61.4–86.5
				Accuracy	73.1	62.3–81.7
				AUC	0.772	0.669–0.875
P-wave duration cutoff 146 ms	Positive	26	11	Sensitivity	74.3	57.9–85.8
	Negative	9	32	Specificity	74.4	59.8–85.1
				PPV	70.3	54.2–82.5
				NPV	78.0	63.3–88.0
				Accuracy	74.4	63.7–82.7
				AUC	0.786	0.684–0.889
DR-FLASH	Positive	19	5	Sensitivity	54.3	38.2–69.5
	Negative	16	38	Specificity	88.4	75.5–94.9
				PPV	79.2	59.5–90.8
				NPV	70.4	57.2–80.9
				Accuracy	73.1	62.3–81.7
				AUC	0.763	0.655–0.871
CHA_2_DS_2_-Va	Positive	15	10	Sensitivity	37.1	23.2–53.7
	Negative	20	33	Specificity	83.7	70.0–91.9
				PPV	65.0	43.3–81.9
				NPV	62.1	49.2–73.4
				Accuracy	62.8	51.7–72.7
				AUC	0.618	0.493–0.743

**Table 3:** Individual component results by majority.

Method		LVA			Value (%)	95% CI
	Test result	Positive	Negative	Metric		
AT	Positive	9	1	Sensitivity	25.7	14.2–42.1
	Negative	26	42	Specificity	97.7	87.9–99.6
				PPV	90.0	59.6–98.2
				NPV	61.8	49.9–72.4
				Accuracy	65.4	54.3–75.0
				AUC	0.617	0.540–0.694
V1	Positive	16	0	Sensitivity	45.7	30.5–61.8
	Negative	19	43	Specificity	100.0	91.8–100.0
				PPV	100.0	80.6–100.0
				NPV	69.4	57.0–79.4
				Accuracy	75.6	65.1–83.8
				AUC	0.729	0.645–0.812
BBBB	Positive	16	1	Sensitivity	45.7	30.5–61.8
	Negative	19	42	Specificity	97.7	87.9–99.6
				PPV	94.1	73.0–99.0
				NPV	68.9	56.4–79.1
				Accuracy	74.4	63.7–82.7
				AUC	0.717	0.630–0.804
PWP	Positive	21	1	Sensitivity	60.0	43.6–74.4
	Negative	14	42	Specificity	97.7	87.9–99.6
				PPV	95.4	78.2–99.2
				NPV	75.0	62.3–84.5
				Accuracy	80.8	70.7–88.0
				AUC	0.788	0.703–0.874
ECG-criteria 2.3.4	Positive	24	1	Sensitivity	68.6	52.0–81.4
ECG-only	Negative	11	42	Specificity	97.7	87.9–99.6
				PPV	96.0	80.5–99.3
				NPV	79.2	66.5–88.0
				Accuracy	84.6	75.0–91.0
				AUC	0.831	0.750–0.913

Of the 78 patients, 49 were mapped in sinus rhythm (31 LVA-negative, 18 LVA-positive) and 29 during CS pacing (12 LVA-negative, 17 LVA-positive). Diagnostic performance of the ECG criteria was consistent across both mapping conditions, with an AUC of 0.901 (95% CI 0.807–0.995) in SR-mapped patients and 0.900 (95% CI 0.786–1.000) in CS pacing-mapped patients. Full diagnostic metrics stratified by mapping rhythm are provided in Supplementary Table 1. In an additional sensitivity analysis restricted to patients with ECGs obtained within 12 months of ablation (*n* = 66), diagnostic performance was consistent with the primary analysis: AUC 0.907 (95% CI 0.835–0.979), sensitivity 87.1% (95% CI 71.1–94.9), specificity 94.3% (95% CI 81.4–98.4), PPV 93.1% (95% CI 78.0–98.1), NPV 89.2% (95% CI 75.3–95.7), and accuracy 90.9% (95% CI 81.6–95.8) (Supplementary Table 1). Exploratory analysis showed no consistent stepwise increase in LVA beyond the first positive criterion (Supplementary Figure 1). On multivariable Firth's penalized logistic regression, ECG criteria majority classification remained the strongest independent predictor of LVAs (*p* < 0.001), alongside persistent AF type (*p* = 0.015), after adjustment for obesity, LVEF, heart failure, LA volume, and antiarrhythmic drug use. No other variables reached statistical significance. Full results are provided in Supplementary Table 2.

### Interobserver variability

Interobserver agreement was substantial, with a Conger's Kappa of 0.653 (95% CI: 0.547–0.760, *p* < 0.001) and observed agreement of 82.7%. Individual observer AUCs ranged from 0.744 to 0.948 (Table 4).

**Table 4:** Test results.

Method		LVA			Value (%)	95% CI
	Test result	Positive	Negative	Metric		
Observer 1	Positive	41	2	Sensitivity	94.3	81.4–98.4
	Negative	2	33	Specificity	95.3	84.5–98.7
				PPV	94.3	81.4–98.4
				NPV	95.3	84.5–98.7
				Accuracy	94.9	87.5–98.0
				AUC	0.948	0.898–0.999
Observer 2	Positive	32	11	Sensitivity	74.3	57.9–85.8
	Negative	9	26	Specificity	74.4	59.8–85.1
				PPV	70.3	54.2–82.5
				NPV	78.0	63.3–88.0
				Accuracy	74.4	63.7–82.7
				AUC	0.744	0.645–0.842
Observer 3	Positive	31	12	Sensitivity	97.1	85.5–99.5
	Negative	1	34	Specificity	72.1	57.3–83.3
				PPV	73.9	59.7–84.4
				NPV	96.9	84.3–99.4
				Accuracy	83.3	73.5–90.0
				AUC	0.846	0.773–0.920
Observer 4	Positive	35	8	Sensitivity	71.4	54.9–83.7
	Negative	10	25	Specificity	81.4	67.4–90.3
				PPV	75.8	59.0–87.2
				NPV	77.8	63.7–87.5
				Accuracy	76.9	66.4–84.9
				AUC	0.764	0.668–0.860
Observer 5	Positive	39	4	Sensitivity	77.1	61.0–87.9
	Negative	8	27	Specificity	90.7	78.4–96.3
				PPV	87.1	71.1–94.9
				NPV	83.0	69.9–91.1
				Accuracy	84.6	75.0–91.0
				AUC	0.839	0.756–0.922

### Performance clinical risk score and P-wave duration

**Figure 7:**
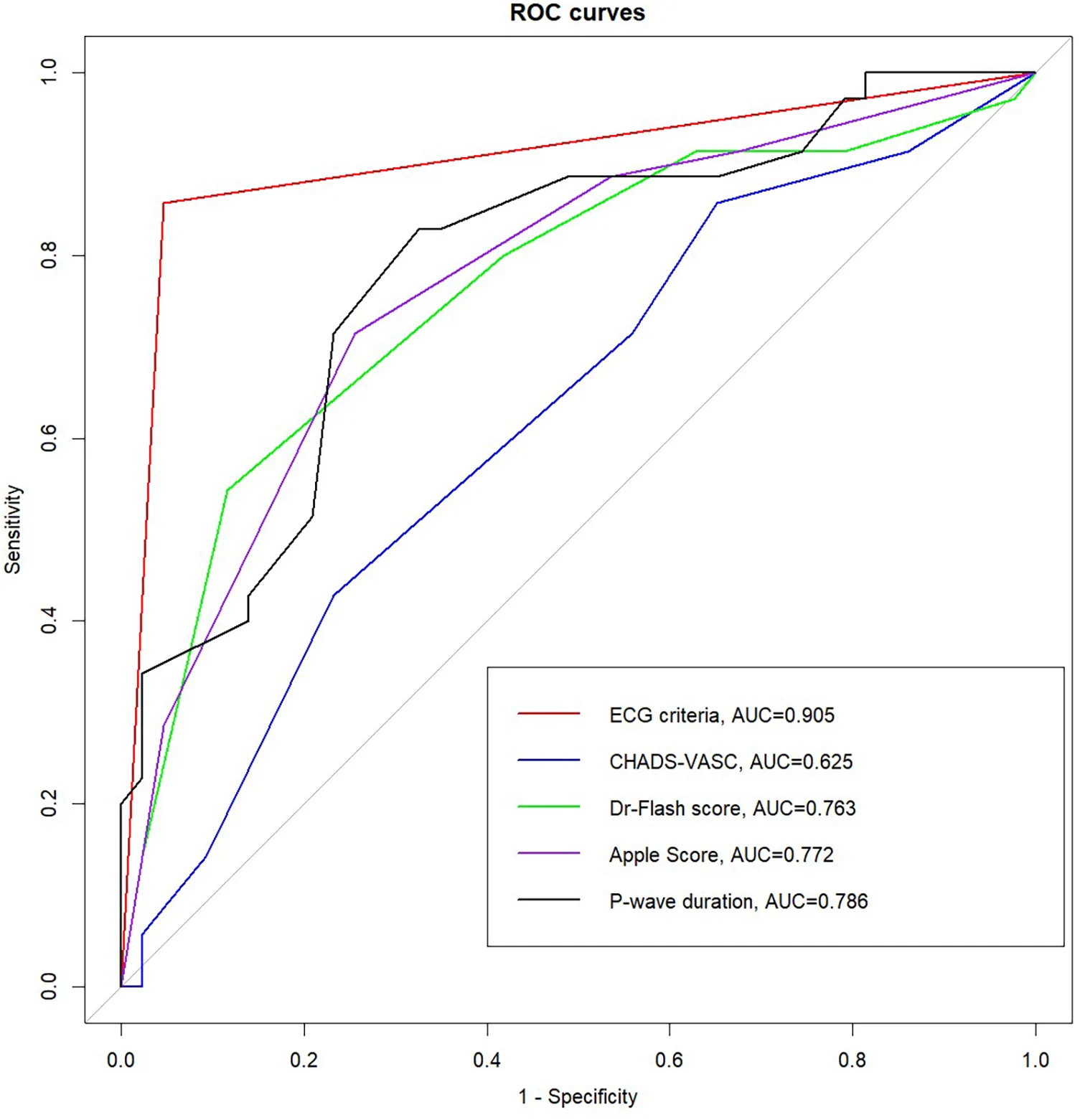
AUC analysis and ROC curves for different screening methods.

The proposed ECG criteria were compared to three different previously described risk scores. The APPLE and DR-FLASH score had similar performance on their optimal cut-off points (AUC 0.772 vs. 0.763), while the CHA_2_DS_2_-VA score performed worse, with an AUC of 0.625. (Table 2, Figure 7). At the optimal cut-off point of 146 ms, P-wave duration performed well with an AUC of 0.786 and accuracy of 74.4%. Sensitivity analysis using alternative cutoff points of 140 and 150 ms demonstrated comparable performance at 140 ms (accuracy 73.1%), whereas a reduction in accuracy was observed at 150 ms (66.7%).

## Discussion

This pilot study suggests that specific, reproducible morphological P-wave features may serve as reliable non-invasive indicators of LVAs. The proposed morphology-based ECG criteria showed a strong association with the presence of LVAs as defined by invasive voltage mapping, while remaining simple, inexpensive, and widely applicable. These findings suggest that the proposed criteria may allow for non-invasive phenotyping of left-atrial LVAs prior to ablation. Whether this information can improve procedural planning or outcomes remains to be seen.

### Physiological basis of the criteria

Criteria 2 and 4 represent novel morphological ECG parameters predefined prior to data analysis. Supporting evidence from the literature was subsequently identified but limited and should be considered convergent rather than the basis for their derivation. Criteria 1 and 3 build upon well-established electrophysiological concepts.

The presence of atypical atrial flutter (criterion 1) reflects advanced atrial remodeling and has been associated with diseased atrial tissue capable of sustaining re-entrant circuits (22, 23). The remaining three criteria evaluate sinus rhythm P-wave morphology from different spatial perspectives.

Lead V1 provides key insight into atrial activation vectors. Under normal conditions, the initial positive component of the P-wave reflects right atrial activation, followed by a terminal negative component corresponding to left atrial depolarization directed posteriorly. In the presence of LVAs, left atrial conduction may become delayed, or reduced, attenuating the terminal negative component in V1 and shifting the balance toward the initial right atrial vector (24). The positive-to-negative amplitude ratio in V1 was introduced to quantify this imbalance. This ratio-based approach may offer advantages over absolute measurements, particularly in conditions where opposing electrophysiological effects, such as atrial dilatation and LVAs, exert partially opposing influences on terminal P-wave morphology (25–28).

Finally, it should be noted that the precordial leads, and especially leads V1–V2, are sensitive to lead placement (29, 30). Although literature on the prevalence of precordial lead misplacement is limited, upward misplacement appears more common than downward, which would increase the terminal negative component and potentially generate false negatives for criterion 2. This underscores that high-quality, standardized ECG acquisition is a prerequisite for reliable morphological assessment, regardless of the parameter evaluated (31).

The third criterion is known as A-IAB and has been first described by Bayés de Luna in 1988. It is now accepted as a separate entity outside of LA enlargement (10, 21). It has been associated with AF and atrial remodeling, particularly involving the Bachmann bundle region in the atrial anterior roof (9, 32).

Poor terminal P-wave progression in leads V4–V6 is conceptually analogous to QRS R-wave progression. In normal atrial activation, the terminal portion of the P wave becomes progressively more positive across the precordial leads. LVAs involving the anterior or lateral regions may attenuate this progression producing flat or negative terminal deflections across multiple lateral leads.

Taken together, the criteria assess atrial activation along multiple spatial axes. The inclusion of atypical atrial flutter may further identify patients with more advanced or clinically manifest substrate abnormalities that are not fully captured by sinus rhythm morphology alone.

### Reproducibility

Interobserver agreement for the detection of LVAs was substantial (Kappa of 0.653, overall agreement 82.7%), even without formal training (33). Individual AUC values ranged from 0.744 to 0.948, reflecting inherent variability near decision thresholds that is expected in composite scoring systems. Reproducibility could be reasonably improved through standardized training protocols or artificial intelligence automated ECG analysis.

The criteria were evaluated using amplified ECG recordings (100 mm/s, 40 mm/mV). While non-standard, modern digital ECG systems typically allow retrospective adjustment of paper speed and amplitude or digital zooming, which may approximate similar visual resolution. Future studies should evaluate the performance of these criteria using standard ECG recordings and assess whether digital post-processing or automated analysis can provide comparable diagnostic accuracy.

### Relation to previous ECG-based predictors of atrial substrate

Several previous studies have explored ECG-based markers of atrial remodeling and LVAs. Amplified P-wave analysis has been shown to localize and estimate the extent of low-voltage substrate, while advanced interatrial block, prolonged P-wave duration, P-wave dispersion and other abnormalities of terminal P-wave morphology have all been associated with atrial remodeling and adverse rhythm outcomes after AF ablation (9, 12, 34–36).

The present study differs from most previously described approaches in that it combines multiple pre-specified morphological features into a simple screening framework. Whereas many ECG-based markers rely on quantitative measurements requiring calipers, amplified recordings, or software-assisted analysis, the proposed criteria were designed to be primarily morphology-based and visually recognizable. The inclusion of two novel criteria, negative atrial vector attenuation in V1 and poor terminal P-wave progression in V4–V6, further expands the spectrum of ECG features potentially associated with atrial substrate abnormality.

These findings suggest that atrial substrate abnormalities may be reflected by a broader spectrum of P-wave morphological changes than traditionally appreciated. The proposed four-criteria framework warrants further evaluation in larger independent cohorts to determine its reproducibility, generalizability, and potential clinical utility.

### Comparison with current risk scores and P-wave duration

Clinical risk scores demonstrate inconsistent discriminative performance for LVAs, reflecting their reliance on indirect clinical surrogates of a multifactorial remodeling process (16). However, the magnitude of these associations varied considerably across studies and subgroups, reflecting heterogeneity in study populations, measurement techniques, and definitions of low-voltage areas. As a result, clinical variables and derived risk scores may demonstrate inconsistent discriminative performance when used as standalone predictors. These findings highlight both the multifactorial nature of atrial remodeling and the limitations of clinical risk scores as indirect surrogates of atrial substrate.

The APPLE, DR-FLASH, and CHA_2_DS_2_-VA scores were evaluated as comparators. The CHA_2_DS_2_-VA score is used to guide the need for long-term anti-coagulation in AF (37). The APPLE score was used as a score to predict AF recurrence after AF catheter ablation (38). However, it has also been tried as a way to predict the presence of low-voltage areas (39). Finally, the DR-FLASH score was specifically developed as a way to evaluate LVAs (18).

The three clinical risk scores performed similarly to previously reported AUC values. The CHA_2_DS_2_-VA score performed poorly (AUC: 0.625), as expected given it was developed for stroke prediction. The APPLE score performed better in our cohort compared to its initial validation performance (AUC: 0.772 vs. 0.711 respectively), possibly driven by higher LVA prevalence (45% vs. 26%) (39). The DR-FLASH score replicated its initial validation performance (AUC 0.763 vs. reported 0.767) (40).

P-wave duration demonstrated good discriminative ability at 146 ms (AUC: 0.786), consistent with previous studies and with the current EHRA consensus statement on atrial cardiomyopathy (41, 42). However, electrocardiographic measurements are inherently subject to measurement error for which a sensitivity analysis was performed. While the performance at 140 ms remained comparable, this was not the case for 150 ms, illustrating sensitivity to small measurement differences. A key gap in the literature is inter-observer variability, which was also explicitly noted in a recent meta-analysis, which should be specifically addressed in future studies.

### Threshold selection and LVA burden

The 5% threshold used to dichotomize LVA burden was pre-specified to approximate MRI-based staging systems, avoiding *post-hoc* optimization bias. While this threshold is acknowledged as somewhat arbitrary in the context of voltage mapping, it proved conservative in practice. LVA-positive patients demonstrated a median low-voltage area of 34.0% with 17 of 35 positive patients classified as >35%, indicating that the majority of positive patients had substantial rather than borderline disease. This limits concern about misclassification around the threshold boundary and supports the clinical relevance of the identified positive cases.

The high observed prevalence reflects selection bias inherent to study design as mapping was performed in patients with suspected substrate abnormalities and the cohort should therefore not be considered representative of an unselected AF ablation population.

No clear dose–response relationship was observed between the number of ECG criteria and LVA prevalence beyond the presence of at least one criterion, suggesting that the criteria primarily function as a binary marker rather than a quantitative measure of disease burden.

### Clinical implications

If validated in larger, multicentric cohorts, the proposed criteria may offer a possibility to determine the presence of LVAs, based on easily retrievable parameters on 12-lead ECG, with a low associated cost.

Notably, the type of AF was not considered in these criteria. While more prevalent in persistent AF, both patients with paroxysmal and persistent AF presented with LVAs. The presented criteria were able to discriminate between normal voltages and LVAs independently of whether AF was paroxysmal or persistent. Whether or not this has clinical implications (e.g., ablation strategies) has to be studied in larger patient cohorts.

### Limitations

This study has several limitations. Its retrospective, single-center design and small sample size limit generalizability. These factors may result in optimistic performance estimates and potential overfitting of the proposed criteria, underscoring the need for validation in independent and larger multicenter cohorts. Spectrum and verification bias are present, as the population consists exclusively of patients referred for AF ablation who underwent invasive mapping, which may not reflect the broader population in whom these criteria would ultimately be applied. Selection bias is introduced by mapping being performed at operator discretion rather than systematically. Voltage maps were acquired using five different catheter types, including both ablation catheters (SmartTouch, QDOT) and high-density mapping catheters (PENTARAY, LASSOSTAR, OCTARAY), reflecting the evolving availability of high-density mapping technology over the study period. These catheter types differ substantially in electrode size, interelectrode spacing, and mapping density, all of which may influence bipolar voltage amplitudes. Furthermore, mapping density varies considerably between catheter types, with sparser maps potentially missing focal areas of low voltage. These factors may have introduced heterogeneity in LVA classification across patients, representing an important limitation of the reference standard. The consistent diagnostic performance observed across both mapping rhythms provides some reassurance, but catheter-specific subgroup analyses were not performed due to insufficient sample sizes within each catheter subgroup and will be addressed in the planned multicenter validation study with pre-specified requirements for high-density mapping. Finally, the threshold used to dichotomize LVA burden (≥5%) is acknowledged as somewhat arbitrary, as no universally accepted cutoff exists in the context of bipolar voltage mapping. Sensitivity analyses using alternative thresholds were not performed given the heterogeneity in mapping catheter types and density, where varying technical conditions risk producing findings that reflect measurement variability rather than true biological differences. Systematic evaluation of alternative thresholds is planned in a multicenter validation study, where pre-specified requirements for high-density mapping will reduce technical heterogeneity and allow more meaningful threshold comparisons.

ECGs were obtained within a variable time window prior to ablation, introducing potential temporal mismatch between ECG findings and atrial substrate and the impact of temporal changes in atrial remodeling on ECG morphology was not formally assessed. However, a sensitivity analysis excluding ECGs obtained more than 12 months prior to ablation demonstrated consistent diagnostic performance, partially mitigating this concern.

Finally, the absence of an independent validation cohort is an important limitation. These findings should be interpreted as hypothesis-generating only, and multicenter validation is needed to confirm reproducibility and determine the utility of these criteria in guiding ablation strategies.

### Future directions

A multicenter validation study is underway to validate this proof-of-concept in a larger cohort with a predetermined minimal amount of voltage points (>1,000 points) on high-density voltage maps. Furthermore, a study has started to evaluate its predictive value for long-term procedural outcomes, particularly in identifying PVI-only responders.

## Conclusions

Morphology-based ECG criteria combining clinical and P-wave morphological features demonstrated promising diagnostic performance in this exploratory cohort. These criteria put forward two well-established parameters correlated with LVAs, and combine them with two conceptual new ECG parameters, “Negative atrial vector attenuation in V1” and “poor terminal P-wave progression in V4–V6”. While diagnostic performance appears promising and may help in phenotyping patients with left atrial substrate, these findings require validation in larger multicenter cohorts before clinical implementation.

## Data Availability

The raw data supporting the conclusions of this article will be made available by the authors upon reasonable request.
